# Decisional Balance for Insulin Injection: Scale Development and Psychometric Testing

**DOI:** 10.1097/jnr.0000000000000316

**Published:** 2019-09-20

**Authors:** Hui-Chun HSU, Shi-Yu CHEN, Yu-Chi HUANG, Ruey-Hsia WANG, Yau-Jiunn LEE, Ling-Wang AN

**Affiliations:** 1 PhD, RN, Certified Diabetes Educator, Department of Diabetes Management, Lee's Endocrinology Clinic, Pingtung, Taiwan, ROC; 2 BSN, RN, Certified Diabetes Educator, Division of Endocrinology & Metabolism, Tri-Service General Hospital, Taipei, Taiwan, ROC; 3 MSc, Casual Research Assistant, University of Melbourne, Melbourne, Australia; 4 PhD, RN, Professor, College of Nursing, Kaohsiung Medical University; and Adjunct Researcher, Department of Medical Research, Kaohsiung Medical University Hospital, Taiwan, ROC; 5 PhD, MD, Department Head, Lee's Endocrinology Clinic, Pingtung, Taiwan, ROC; 6 MD, Director, Department of Medical Education, Beijing Ruijing Diabetes Hospital, Beijing, China.

**Keywords:** insulin injection, decisional balance, transtheoretical model, type 2 diabetes mellitus

## Abstract

**Background:**

Insulin-naive patients are often reluctant to receive insulin treatment, and even insulin-treated patients frequently have poor rates of adherence to their prescribed insulin injection regimes. Assessing attitudes toward insulin injection may help in the design of interventions that improve the insulin injection behaviors of patients with type 2 diabetes (T2DM). The concept of decisional balance has been associated with behavior in many studies and may be useful in assessing the attitude of patients with T2DM toward insulin injection. Decisional balance for insulin injection (DBII) has not been widely assessed in patients with T2DM.

**Purpose:**

The aim of this study was to develop an insulin injection (DBII) scale that is valid for insulin-naive and insulin-treated patients and to test the psychometric characteristics of this scale based on the concept of decisional balance.

**Methods:**

This cross-sectional study administered an 18-item DBII scale, including pro and con subscales, to 95 insulin-naive and 237 insulin-treated patients in Taiwan. The decisional balance score was calculated as the mean score of the pro subscale minus the mean score of the con subscale. Construct validity was examined using exploratory factor analysis and confirmatory factor analysis; concurrent validity was assessed by examining the association between the score of the DBII scale and the stages of behavioral change and of hemoglobin A1c for, respectively, insulin-naive patients and insulin-treated patients; and reliability was assessed using internal consistency and test–retest reliability.

**Results:**

A 13-item DBII scale supported by exploratory factor analysis and confirmatory factor analysis was developed. The stages of behavioral change and hemoglobin A1c levels were found to be significantly associated with the scores of decisional balance of the 13-item DBII scale for both insulin-naive and insulin-treated patients. The Cronbach's α ranged between .78 and .92.

**Conclusions:**

The 13-item DBII scale is appropriately short and possesses satisfactory validity and reliability for both insulin-naive and insulin-treated patients with T2DM. Healthcare providers may use this scale as a checklist to guide clinical discussions related to insulin therapy with both insulin-naive and insulin-treated patients with T2DM across time.

## Introduction

Diabetes is a prevalent chronic disease in many developed countries. Approximately one in 10 adults is affected by diabetes worldwide, which represents around 415 million individuals (International Diabetes Federation, 2017). It has been estimated that, by the year 2040, 642 million individuals will have type 2 diabetes mellitus (T2DM) globally (International Diabetes Federation, 2017). In Taiwan, the prevalence of diabetes has risen to 9.2% and was the fifth leading cause of death in 2016 (Department of Statistics, Ministry of Health and Welfare, Taiwan, ROC, 2017). Poor diabetes control often results in serious macrovascular and microvascular complications such as coronary artery disease and neuropathy (Nichols, Rosales, Perrin, & Fortmann, 2014). Thus, controlling T2DM is an urgent international public health issue.

Traditionally, patients are initially treated with oral hypoglycemic agents after receiving a T2DM diagnosis. Nevertheless, T2DM is a progressive disease, and almost 80% of people with T2DM will require insulin to maintain optimal hemoglobin A1c (HbA1c) levels (Hermansen, Mortensen, & Hermansen, 2008). Insulin therapy is suggested for patients with T2DM being treated with oral hypoglycemic agents but who report poor glycemic control (American Diabetes Association, 2018). However, many patients with T2DM being treated with oral hypoglycemic agents and reporting poor glycemic control are often reluctant to receive insulin treatment (Lee, 2015; Polonsky, Hajos, Dain, & Snoek, 2011). Even insulin-treated patients may have poor adherence to insulin injection (Davies et al., 2013; Peyrot, Barnett, Meneghini, & Schumm-Draeger, 2012). As attitude has been identified as an important determinant of behavior in many behavioral models (Ajzen, 1991; Bamberg, 2013), assessing attitude toward insulin injection is crucial for healthcare providers to improve the initiation of as well as adherence to insulin injection regimens.

Fear of weight gain, needle injection, pain, hypoglycemia, public embarrassment, and interference with daily life have been identified as prevalent, negative attitudes toward insulin injection (Farsaei, Radfar, Heydari, Abbasi, & Qorbani, 2014; Peyrot et al., 2012; Polinski et al., 2013), whereas prevention of complications, maintenance of good glycemic control, improved energy levels, and family support have been identified as facilitators of insulin injection acceptance (Davies et al., 2013; Holmes-Truscott, Pouwer, & Speight, 2014; Peyrot et al., 2012). Most of the scales currently used to measure attitudes toward insulin injection have focused on assessing related negative attitudes or perceived barriers, which are collectively referred to as psychological insulin resistance and are specific to “insulin-naive” (no prior insulin use) patients with T2DM (Brod, Alolga, & Meneghini, 2014; Woudenberg, Lucas, Latour, & Scholte op Reimer, 2012). For example, the 14-item Barriers to Insulin Treatment Scale (BITS) has been used widely to measure perceived barriers of initial insulin injection in insulin-naive patients (Petrak et al., 2007). The BITS includes factors such as attitudes toward fear of injection and self-testing, expectations regarding positive insulin treatment, expected hardships of insulin treatment, stigmatization of insulin injections, and fear of hypoglycemia. The factor structure of BITS has been supported by confirmatory factor analysis (CFA), and the overall Cronbach's α is .78. However, BITS items are rated from 1 to 10, which may not be convenient for patients. In addition, a 13-item Chinese Attitudes to Starting Insulin Questionnaire (Ch-ASIQ) was developed for Chinese-speaking populations as a measure of self-image and stigmatization, fear of pain or needles, factors promoting self-efficacy, and time and family support (Fu et al., 2013). Despite this questionnaire earning Cronbach's α values for each factor of ≥ .60, its construct validity was only supported by exploratory factor analysis (EFA), and no further validity measures were used.

Psychological insulin resistance has been associated with poor adherence to insulin injection in insulin-treated patients (Yavuz, Ozcan, & Deyneli, 2015). Nevertheless, few scales have been developed specifically to assess attitude toward insulin injection in insulin-treated patients. The 20-item Insulin Treatment Appraisal Scale (ITAS) was designed to assess negative attitudes toward insulin injection in insulin-naive and insulin-treated patients (Snoek, Skovlund, & Pouwer, 2007) and earned a Cronbach's α of .89, indicating satisfactory internal consistency. The concurrent validity of the ITAS, which combined insulin-naive and insulin-treated patients, was supported by a low-to-moderate correlation with emotional distress and well-being. Two factors were found to best support the latent structure of ITAS after EFA. Moreover, the ITAS scores of insulin-naive patients were significantly higher than those of insulin-treated patients in the United States (Snoek et al., 2007), Australia (Holmes-Truscott et al., 2014), and China (Chen et al., 2011), which preliminarily supports the known-group validity of ITAS. Nevertheless, the validity of ITAS in populations of insulin-naive and insulin-treated patients has not been examined.

Many scales, including BITS, Ch-ASIQ, and ITAS, focus on measuring negative attitudes toward insulin injection. Even when positive-attitude items are designed into these scales, they are reverse scored and summed with the scores of negative-attitude items to quantify an overall score of negative appraisal for insulin injection. Assessing only the negative attitudes toward insulin injection may lead healthcare providers to focus on reducing barriers and to overlook the potential facilitators of improving insulin injection efficacy. Holmes-Truscott, Pouwer, and Speight (2017) asserted that positive attitudes toward insulin injection merited more assessment to educate patients comprehensively. The transtheoretical model (TTM; Prochaska, Redding, & Evers, 2008) proposed behavioral change as an ongoing process rather than as a discrete occurrence. According to the TTM, individuals experience behavioral stages of precontemplation (no intention to take action in the coming 6 months), contemplation (intention to act within 6 months), preparation (intention to act within a month), action (already modified behavior within the past 6 months), and, finally, maintaining the behavior (already adopted a behavioral change for over 6 months and are thus less likely to relapse). The TTM proposed “decisional balance” to be an attitude factor that is significantly associated with behavioral stages (Prochaska et al., 2008). Decisional balance refers to people who consider pros and cons simultaneously and assess the relative impact of both before adopting positive behavioral stages. The cons outweigh the pros in the precontemplation stage, whereas the pros outweigh the cons in the contemplation, preparation, and action stages. The concept of decisional balance has been found to associate with behaviors and health outcomes in many studies (Shtaynberger & Krebs, 2016; Therawiwat, Imamee, & Khamklueng, 2013). Although the concept of the decisional balance of TTM may be useful in assessing attitude toward insulin injection, this has been less tested for insulin injection in patients with T2DM.

Considering developing a scale to measure both positive and negative attitudes toward insulin injection simultaneously may help healthcare providers develop comprehensive care to improve insulin injection efficacy in patients with T2DM. Furthermore, a scale that is applicable for insulin-naive and insulin-treated patients may help track the progress of these patients from insulin-naive to insulin-treated status. Therefore, the aim of this study was to develop a decisional balance for insulin injection (DBII) scale that is valid for use with insulin-naive and insulin-treated patients and to test the psychometric characteristics of this scale using the concept of decisional balance.

## Methods

### Sampling and Design

A cross-sectional design was used in this study. Considering that DBII is applicable to both insulin-naive and insulin-treated patients, patients in both categories were recruited for this study. Insulin-naive and insulin-treated patients with T2DM were recruited using convenience sampling from two outpatient diabetes clinics in Taiwan. All eligible participants were between 20 and 85 years old and had been diagnosed with T2DM for at least 6 months. The inclusion criteria for insulin-naive patients were as follows: (a) taking oral hypoglycemic agents and having no previous experience of insulin injection and (b) having HbA1c levels higher than 8.5% more than two times during the most recent 1-year period while eligible for initiating insulin injection (Monnier, Lapinski, & Colette, 2003). The inclusion criterion for insulin-treated patients was having insulin treatment for at least 3 months. Endocrine physicians referred eligible participants to a research assistant. After the research assistant explained the study purposes and eligible participants signed consent forms, an anonymous questionnaire, including the first draft of the 18-item DBII scale, and a demographic and disease characteristics datasheet were administered. Overall, 332 patients were recruited for this study. Data were collected from November 2016 to September 2017.

The detailed procedures that were used to develop the DBII scale and to test its psychometric properties are described below.

#### Development of the first draft of the decisional balance for insulin injection scale and content validity testing

A first draft of the 18-item DBII scale was developed based on previously developed scales such as the BITS, Ch-ASIQ, and ITAS as well as on related articles in the literature (Davies et al., 2013; Farsaei et al., 2014; Fu et al., 2013; Holmes-Truscott et al., 2014; Petrak et al., 2007; Peyrot et al., 2012; Polinski et al., 2013; Snoek et al., 2007). Moreover, this first draft included pro and con subscales based on the concept of decisional balance in the TTM. The con subscale included 10 items related to perceived inconvenience, fear of pain, poor skill, and the perceived negative consequences of insulin injection, whereas the pro subscale included eight items related to perceived convenience, family support, and the positive consequences of insulin injection. Each item was rated from 1 (*strongly disagree*) to 5 (*strongly agree*). Mean item scores of the two subscales were calculated separately. High item mean scores on the pro subscale indicated a more positive attitude toward insulin injection, whereas higher item mean scores on the con subscale indicated a more negative attitude toward insulin injection. The item mean score of the pro subscale minus the item mean score of the con subscale was calculated to represent the score of DBII. The total possible score ranged from −4 to 4, with higher scores indicating a stronger positive attitude toward insulin injection.

Two diabetes physicians and five diabetes nurses were invited to rate the relevance of each item on the draft DBII using a scale ranging from 1 (*irrelevant*) to 4 (*highly relevant and succinct*). The content validity index was calculated by dividing the number of items that were rated 3 or 4 by the total number of items, giving a content validity index value for the draft DBII scale of .87, which indicates that this scale is acceptable for further use (Waltz, Strickland, & Lenz, 1991).

#### Item analysis and construct validity testing by exploratory factor analysis and confirmatory factor analysis

The DBII scale was developed to be applicable for patients with T2DM regardless of insulin treatment status. Therefore, the data of insulin-naive and insulin-treated patients were combined together to conduct item analysis and construct validity. Item analysis was conducted to delete redundant items. Items with item–total correlations below .4 are deemed to not sufficiently contribute to measuring the concept, whereas those above .7 may be redundant (Ferketich, 1991). Therefore, items with an item–total correlation below .4 or above .7 were considered for deletion. The items that were retained after item analysis were analyzed to examine construct validity.

Construct validity was initially tested using EFA. The factor structure produced by EFA was further cross-validated using CFA. Principal component analysis with oblique rotation was conducted to perform EFA, as the pro and con scores should be correlated. Factors with eigenvalues exceeding unity were used to select the factors to be rotated. Having a factor loading larger than .5 was set as the criterion for retaining items in the DBII scale (Gorsuch, 1997). Structural equation modeling was performed to conduct CFA. CFA was considered acceptable if the χ^2^/*df* ratio was lower than 3. The goodness-of-fit index (GFI), comparative fit index (CFI), and nonnormed fit index (NNFI) all exceeded .90, and the root mean square error of approximation (RMSEA) was less than .08 (Ullman, 2006). The final version of DBII was developed after EFA and CFA were completed.

#### Known-group validity testing

Known-group validity is the ability to distinguish the differences between specific groups of individuals that are anticipated to exhibit different scores (Netemeyer, Bearden, & Sharma, 2003). Insulin-naive patients have been found to hold more negative attitudes toward insulin than insulin-treated patients (Chen et al., 2011; Holmes-Truscott et al., 2014). Therefore, comparing the pro scores, con scores, and decisional balance scores between insulin-naive and insulin-treated patients was conducted in this study to examine the known-group validity of the final DBII scale.

#### Concurrent validity testing

The concurrent validity of insulin-naive and insulin-treated patients, respectively, was tested using different criteria indicators. Because insulin-naive patients have not received insulin injection, the DBII of this group should associate with the stages of behavioral change for insulin injection (excluding the stage of action and maintenance) based on TTM (Prochaska, 1994). Therefore, the concurrent validity of the final DBII scale for insulin-naive patients was examined by testing the associations between pro, con, and decisional balance scores, respectively, with the stages of behavioral change for insulin injection. TTM clearly defines stages of behavioral change, with one item frequently used to measure stages of behavioral change (Guicciardi et al., 2014; Holmen et al., 2016). The question “What would you think if the doctor recommended that you initiate insulin treatment right now?” was developed. The three possible responses of “never considered,” “under consideration and may start within the next 6 months,” and “likely to start immediately” were designed to respectively represent three early stages of behavioral change, namely, precontemplation, contemplation, and preparation.

For insulin-treated patients, the final goal of insulin injection is to achieve optimal glycemic control. Therefore, HbA1c level was selected as the criterion to assess the concurrent validity of the final DBII scale. The associations between the pro, con, and decisional balance scores and HbA1c levels were examined. The updated HbA1c was collected from medical records for each insulin-treated patient after the DBII scale had been administered.

#### Reliability testing

Cronbach's α was calculated to assess the internal consistency of the pro and con subscales for all participants and was examined separately in insulin-naive and insulin-treated patients.

### Ethical Considerations

This study was approved by the Human Experiment and Ethics Committee of two hospitals (Nos. KMUHI-E(I)-20150262 and TSGHIRB-1-105-05-163). Researchers explained the study purpose and informed participants of their right to refuse to participate. Data were collected only after the participants had signed the consent forms.

### Statistical Analysis

Item analysis, Cronbach's α, descriptive statistics, group comparison, bivariate correlation, and EFA were performed using SPSS Statistics Version 17.0 (SPSS, Inc., Chicago, IL, USA). CFA was performed using the IBM SPSS Amos Version 18.0 structural equation modeling program (IBM, Armonk, NY, USA). All of the statistical analyses were two sided, and a *p* < .05 was considered significant.

## Results

### Demographic and Disease Characteristics of the Participants

Of the 332 participants, 95 were insulin naive and 237 were insulin treated. Table 1 shows the demographic and disease characteristics for all of the participants and comparisons of these characteristics between the groups. Neither gender nor age differed significantly between the two groups. In Taiwan, most patients with T2DM are first treated with oral medication and are referred for insulin injection treatment if they exhibit poor glycemic control. The duration of diabetes in all insulin-naive patients was significantly lower than that in all insulin-treated patients. As insulin-naive patients were selected for poor glycemic control, their HbA1c levels were significantly higher than those of insulin-treated patients.

**TABLE 1.**
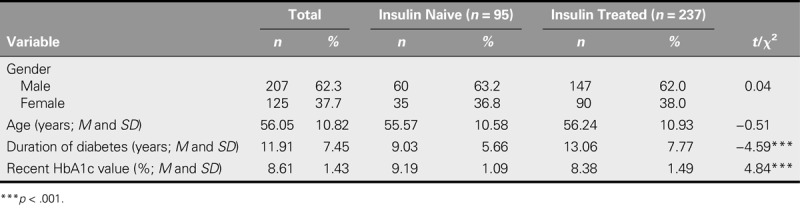
Demographic and Disease Characteristics of Participants and Comparison Between Insulin-Naive and Insulin-Treated Patients (*N* = 332)

### Item Analysis, Exploratory Factor Analysis, and Confirmatory Factor Analysis

After item analysis, five items were deleted because their item–total correlation coefficients were smaller than .4. The construct validity of the retained 13 items was assessed using EFA and CFA. To prevent overfit due to conducting EFA and CFA on the same samples, 332 participants were randomly assigned into Group 1 (*n* = 159) and Group 2 (*n* = 173) by computer to conduct the EFA and CFA, respectively. In terms of EFA, the Kaiser–Meyer–Olkin measure of sampling adequacy was .865 and Bartlett's test of sphericity was with χ^2^ = 1020.42, *p* < .001, which supported the adequacy of this sample to perform EFA. After EFA, the factor loadings of the 13 retained items were all significant and larger than .5. Two factors, named “pros” (eight items) and “cons” (five items), were produced, which explained 63.48% of the total variance (Table 2). No items were cross-loaded on different factors.

**TABLE 2.**
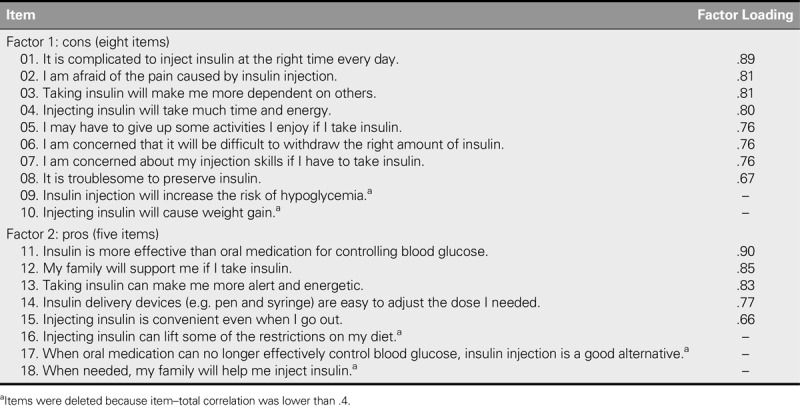
Exploratory Factor Analysis of the Decision Balance for Insulin Injection Scale

**Figure 1.**
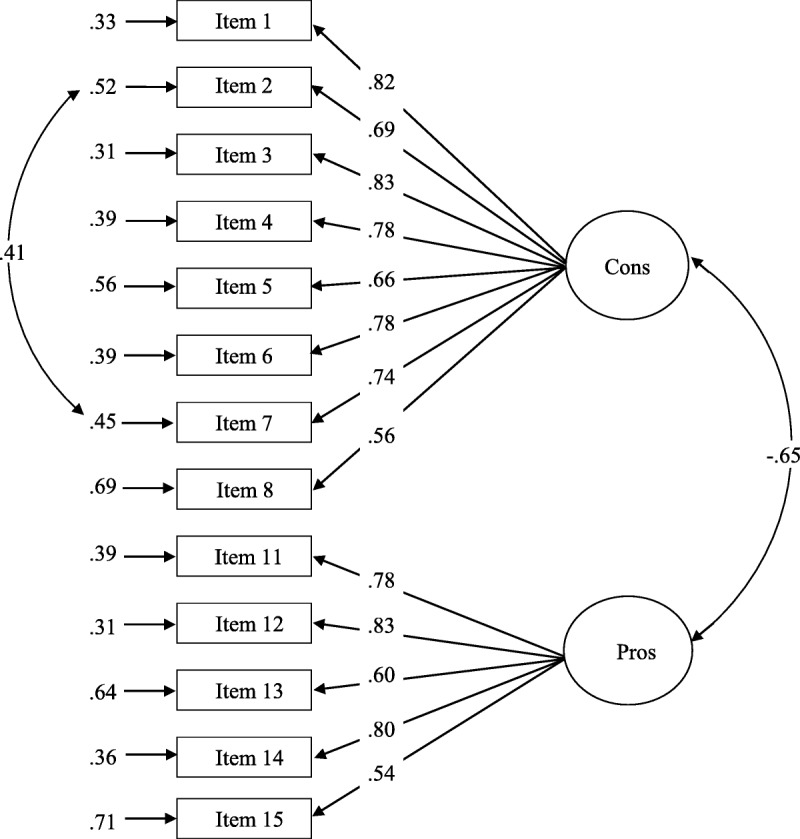
The final first-order confirmatory factor analysis of the 13-item decisional balance for insulin injection scale.

The factor structure produced by EFA was further examined using CFA on Group 2. A two-correlated, first-order CFA was performed to test the factor structure of the 13-item DBII scale. The fit indices were χ^2^ = 159.022, *df* = 64, *χ*^2^/*df* = 2.49, GFI = .88, NNFI = .90, CFI = .92, and RMSEA = .09 (90% CI [0.08, 0.11]). Furthermore, a high correlation between the residuals of Items 2 and 7 was found, according to the modification indices. The correlation of error variances between Items 2 and 7 was set, and CFA was conducted again. As shown in Figure 1, each item significantly loaded on its corresponding factor and factor loadings were all above .5. The fit indices were acceptable, with χ^2^ = 133.01, *df* = 63, *p* < .001, χ^2^/*df* ratio = 2.11, GFI = .90, NNFI = .93, CFI = .94, and RMSEA = .08 (90% CI [0.061, 0.099]). The correlation coefficient between the two first-order factors was −.65. Therefore, the factor structure of the 13-item DBII scale was supported.

### Known-Group Validity and Concurrent Validity

Pros, cons, and DBII were all considered when assessing the known-group validity and concurrent validity of the 13-item DBII scale in insulin-naive and insulin-treated patients, respectively. In terms of known-group validity, the item mean scores of the insulin-naive patients for decisional balance and the pro subscale were significantly lower than those of the insulin-treated patients, whereas the item mean scores of the insulin-naive patients for the con subscale were significantly higher than those of the insulin-treated patients (Table 3).

**TABLE 3.**
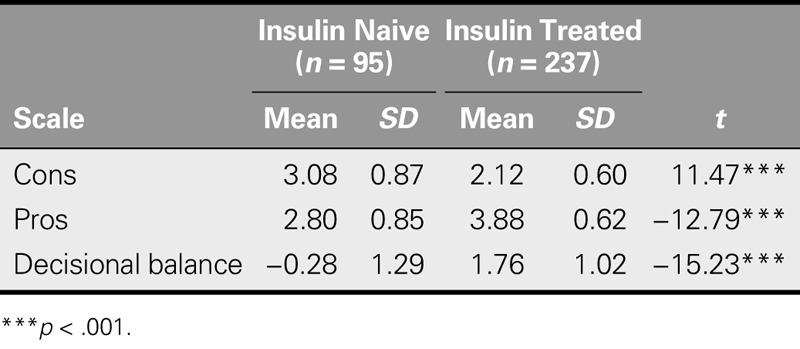
Comparison of Decision Balance for Insulin Injection Between Insulin-Naive and Insulin-Treated Patients With Type 2 Diabetes (*N* = 332)

With respect to concurrent validity, item mean scores for pros, cons, and decisional balance significantly differed for insulin-naive participants among the three stages of behavioral change (Table 4). For this subgroup, the item mean score on the pro subscale in the precontemplation stage was significantly less than those in the contemplation and preparation stages, and that in the contemplation stage was significantly lower than that in the preparation stage. As for decisional balance, the item mean scores for both the contemplation and preparation stages were significantly higher than that for the precontemplation stage. No significant difference was found in item mean scores of the con subscale between any two stages of behavioral stages after post hoc analysis. As for concurrent validity for the insulin-treated patients, the correlation coefficients of the pro subscale, con subscale, and decisional balance with HbA1c levels were −.11 (*p* = .10), .21 (*p* = .001), and −.19 (*p* = .004), respectively.

**TABLE 4.**
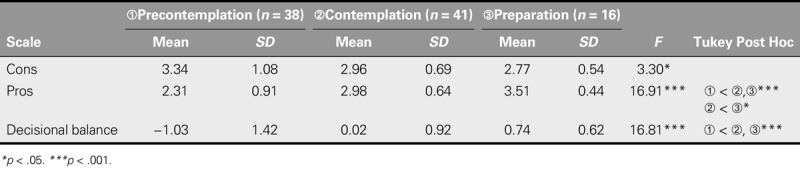
Comparison of Stages of Behavioral Change on Decisional Balance for Insulin Injection in Insulin-Naive Patients With Type 2 Diabetes (*N* = 95)

### Reliability

The Cronbach's α of the pro and con subscales were .87 and .92, respectively. In terms of the Cronbach's α for the insulin-naive patient group only, the values for the pro and con subscales were .86 and .92, respectively. In terms of Cronbach's α for the insulin-treated patient group only, values for the pro and con subscales were .78 and .88, respectively.

## Discussion

As far as is known by the authors, this study is the first to develop a scale to assess patient attitudes toward insulin injection based on the concept of decisional balance and to examine the psychometric characteristics of this scale. The findings of this study support using decisional balance to assess attitudes toward insulin injection in patients with T2DM, regardless of insulin-treatment status.

In this study, items that addressed increased risk of hypoglycemia (Item 9) and weight gain (Item 10) after insulin injection were deleted after item analysis. Similarly, a prior study found that, in the 20-item ITAS, which was designed to be applicable to both insulin-naive and insulin-treated patients, items pertaining to weight gain and increased risk of hypoglycemia had relatively lower factor loadings than other items (Snoek et al., 2007). In addition, previous studies support the finding of this study that Chinese patients appear to be more concerned about the psychosocial aspects than the physical aspects (e.g., risk of hypoglycemia and weight gain) of insulin therapy (Ho & James, 2006; Wang & Yeh, 2012). Participants in this study were not particularly concerned regarding risks of hypoglycemia and weight gain after insulin injection. Nevertheless, further studies are needed to validate this suggestion. Because diet control is emphasized in patients with T2DM regardless of insulin treatment status (Barratt, Frost, Millward, & Truby, 2008), patients may not perceive decreasing diet restrictions as an important benefit of insulin injection. Therefore, Item 16, which addressed the relief of diet restrictions after insulin injection, was not an important indicator of the DBII scale. The content measured in Items 17 (“When oral medication no longer effectively controls blood glucose, insulin injection is a good alternative”) and 18 (“When needed, my family will help me inject insulin”), respectively, overlapped with those of Items 11 (“Insulin is more effective than oral medication for controlling blood glucose”) and 12 (“My family will support me if I take insulin”). Therefore, Items 17 and 18 were deleted after item analysis. The retained items of the 13-item DBII scale were similar to BITS, Ch-ASIQ, and IATS, indicating that the attitudes toward insulin injection of patients with T2DM are similar across different populations.

The construct validity of the 13-item DBII scale was supported by EFA and CFA. In addition, the absolute value of the correlation coefficient between the pro and con subscales (.65) was smaller than .85, indicating good discrimination between these two subscales (Brown, 2015). Thus, the 13-item DBII scale, including the pro and con subscales, was found to be adequate. Significant differences in the scores of decisional balance, pros, and cons were found between insulin-naive and insulin-treated patients, which is consistent with previous studies (Chen et al., 2011; Holmes-Truscott et al., 2014). The 13-item DBII scale distinguished between the attitudes toward insulin injection of insulin-naive and insulin-treated patients and supported the known-group validity of the 13-item DBII scale.

The concurrent validity of the 13-item DBII scale was supported for insulin-naive patients, as the scores of decisional balance significantly associated with the stages of behavioral change. The decisional balance score for the precontemplation stage was significantly lower than those for both the contemplation and preparation stages. The higher the decisional balance scores, the more positive the stages of behavior. Reviewing the scores of the pro and con subscales, the scores of con subscales between any two stages did not differ significantly. However, the pro subscale score seemed to have a dose–response relationship and to increase from the precontemplation stage to the preparation stage. This may indicate that, for insulin-naive patients who were in the precontemplation stage, enhancing the pros and then increasing the decisional balance may help them move from the precontemplation stage to the contemplation stage and then to the preparation stage. Thus, this finding supports the TTM statement that emphasizing the pros of behaviors in the early stage may help patients move to the positive stage of behaviors (Prochaska et al., 2008). Contrary to the traditional approach, which works to decrease negative attitudes toward insulin injection for insulin-naive patients, working to enhance the pros and then to increase decisional balance may be useful to help insulin-naive patients initiate insulin injection.

The concurrent validity of the 13-item DBII scale in insulin-treated patients was supported because higher decisional balance scores were associated with lower HbA1c levels. The more the pros outweighed the cons, the lower the HbA1c levels in insulin-treated patients with T2DM. Furthermore, con subscale scores were found to correlate significantly with HbA1c levels, whereas pro subscale scores did not. Insulin-treated patients may hold positive attitudes toward insulin injection and toward receiving insulin injections. Nevertheless, they may still encounter negative experiences regarding insulin injection and thus possess negative attitudes toward insulin injection (Tong, Vethakkan, & Ng, 2015). Therefore, the cons of insulin injection appear to have a stronger association than the pros with HbA1c levels in insulin-treated patients. Strategies that are designed to enhance decisional balance by decreasing the cons may improve glycemic control in insulin-treated patients. Nevertheless, studies should assess the correlation of the 13-item DBII scale with insulin injection behaviors in insulin-treated patients to examine the concurrent validity of the 13-item DBII scale in clinical settings.

The Cronbach's αs of the pro and con subscales of the 13-item DBII scale for all participants as well as for the insulin-naive and insulin-treated groups were all larger than .7, which is considered acceptable (Rosner, 2006). The 13-item DBII scale showed acceptable reliability independent of insulin treatment status as well as when used exclusively on insulin-naive and insulin-treated patients, respectively.

### The Limitations of This Study

Several limitations affected this study. The 13-item DBII scale was only developed based on the author's review of related literature. Qualitative interviews should be conducted to more comprehensively capture the pros and cons of insulin injection. In addition, the participants were recruited using convenience sampling from only two diabetes clinics in Taiwan. More men and younger-aged patients were included in this study, as compared with a national survey of patients with T2DM in Taiwan (Yu et al., 2013) in which the ratio of male patients was 49.9% and the mean age was 63.2 years (*SD* = 12.2). Thus, the generalizability of this study may be limited. Furthermore, attitudes toward insulin injection may be significantly affected by culture and ethnicity (Wang & Yeh, 2012). Therefore, the 13-item DBII scale must be further cross-validated in diverse populations in Taiwan and in other countries. Further studies should also track the change in the scores of the 13-item DBII scale on the same patients from before to after they receive insulin injections and examine the sensitivity to the change from insulin-naive to insulin-treated status.

### Conclusions and Clinical Implications

As we know, the 13-item DBII scale is the first scale to assess attitudes toward insulin injection based on the concept of decisional balance. The 13-item DBII scale is not only appropriately short but also possesses satisfactory validity and reliability for insulin-naive and insulin-treated patients with T2DM. Healthcare providers such as nurses may use the 13-item DBII scale as a checklist to guide clinical discussions with patients about insulin therapy as they transition from insulin-naive to insulin-treated status over time. Furthermore, this study provides useful information to help improve the DBII for both insulin-naive and insulin-treated patients with T2DM. Healthcare providers may emphasize the importance of increasing the pros for insulin-naive patients and of decreasing the cons for insulin-treated patients.

## References

[bib1] Ajzen I. (1991). The theory of planned behavior. *Organizational Behavior and Human Decision Processes*, 50(2), 179–211. 10.1016/0749-5978(91)90020-T

[bib2] American Diabetes Association. (2018). Improving care and promoting health in populations: Standards of medical care in diabetes—2018. *Diabetes Care*, 41(1, Suppl), S7–S12. 10.2337/dc18-S001 29222372

[bib3] Bamberg S. (2013). Changing enviromentally harmful behaviors: A stage model of self-regulated behavioral change. *Journal of Enviromental Psychology*, 34, 151–159. 10.1016/j.jenvp.2013.01.002

[bib4] Barratt R. Frost G. Millward D. J. & Truby H. (2008). A randomised controlled trial investigating the effect of an intensive lifestyle intervention v. standard care in adults with type 2 diabetes immediately after initiating insulin therapy. *British Journal of Nutrition*, 99(5), 1025–1031. 10.1017/S0007114507839018 18197995

[bib5] Brod M. Alolga S. L. & Meneghini L. (2014). Barriers to Initiating Insulin in type 2 diabetes patients: Development of a new patient education tool to address myths, misconceptions and clinical realities. *Patient*, 7(4), 437–450. 10.1007/s40271-014-0068-x 24958464 PMC4240906

[bib6] Brown T. A. (2015). *Confirmatory factor analysis for applied research* (2nd ed.). London, England: Guilford.

[bib7] Chen C. C. Chang M. P. Hsieh M. H. Huang C. Y. Liao L. N. & Li T. C. (2011). Evaluation of perception of insulin therapy among Chinese patients with type 2 diabetes mellitus. *Diabetes & Metabolism*, 37(5), 389–394. 10.1016/j.diabet.2010.12.008 21458350

[bib8] Davies M. J. Gagliardino J. J. Gray L. J. Khunti K. Mohan V. & Hughes R. (2013). Real-world factors affecting adherence to insulin therapy in patients with type 1 or type 2 diabetes mellitus: A systematic review. *Diabetic Medicine*, 30(5), 512–524. 10.1111/dme.12128 23323988

[bib9] Department of Statistics, Ministry of Health and Welfare, Taiwan, ROC. (2017). *2016 statistics of causes of death*. Retrieved from http://www.mohw.gov.tw/lp-3327-2.html

[bib10] Farsaei S. Radfar M. Heydari Z. Abbasi F. & Qorbani M. (2014). Insulin adherence in patients with diabetes: Risk factors for injection omission. *Primary Care Diabetes*, 8, 338–345. 10.1016/j.pcd.2014.03.001 24721139

[bib11] Ferketich S. (1991). Focus on psychometrics. Aspects of item analysis. *Research in Nursing & Health*, 14(2), 165–168. 10.1002/nur.4770140211 2047538

[bib12] Fu S. N. Chin W. Y. Wong C. K. H. Yeung V. T. F. Yiu M. P. Tsui H. Y. & Chan K. H. (2013). Development and validation of the Chinese attitudes to starting insulin questionnaire (Ch-ASIQ) for primary care patients with type 2 diabetes. *PLoS One*, 8(11), e78933 10.1371/journal.pone.0078933 24236071 PMC3827341

[bib13] Gorsuch R. L. (1997). Exploratory factor analysis: Its role in item analysis. *Journal of Personality Assessment*, 68(3), 532–560. 10.1207/s15327752jpa6803_5 16372866

[bib14] Guicciardi M. Lecis R. Anziani C. Corgiolu L. Porru A. Pusceddu M. & Spanu F. (2014). Type 2 diabetes mellitus, physical activity, exercise self-efficacy, and body satisfaction. An application of the transtheoretical model in older adults. *Health Psychology and Behavioral Medicine*, 2(1), 748–758. 10.1080/21642850.2014.924858 25750816 PMC4346010

[bib15] Hermansen K. Mortensen L. S. & Hermansen M. L. (2008). Combining insulin with oral antidiabetic agents: Effect on hyperglycemic control, markers of cardiovascular risk and disease. *Vascular Health and Risk Management*, 4(3), 561–574. 10.2147/VHRM.S1815 18827907 PMC2515417

[bib16] Ho E. Y. & James J. (2006). Cultural barriers to initiating insulin therapy in Chinese people with type 2 diabetes living in Canada. *Canadian Journal of Diabetes*, 30(4), 390–396. 10.1016/S1499-2671(06)04004-4

[bib17] Holmen H. Wahl A. Torbjørnsen A. Jenum A. K. Småstuen M. C. & Ribu L. (2016). Stages of change for physical activity and dietary habits in persons with type 2 diabetes included in a mobile health intervention: The Norwegian study in RENEWING HEALTH. *BMJ Open Diabetes Research & Care*, 4(1), e000193 10.1136/bmjdrc-2016-000193 PMC4873947 27239317

[bib18] Holmes-Truscott E. Pouwer F. & Speight J. (2014). Further investigation of the psychometric properties of the Insulin Treatment Appraisal Scale among insulin-using and non-insulin-using adults with type 2 diabetes: Results from Diabetes MILES-Australia. *Health and Quality of Life Outcomes*, 12(1), 87–95. 10.1186/1477-7525-12-87 24902877 PMC4050399

[bib19] Holmes-Truscott E. Pouwer F. & Speight J. (2017). Assessing psychological insulin resistance in type 2 diabetes: A critical comparison of measures. *Current Diabetes Reports*, 17(7), 46 10.1007/s11892-017-0873-4 28508930

[bib20] International Diabetes Federation. (2017). *IDF diabetes atlas 8th edition*. Retrieved from http://www.diabetesatlas.org/

[bib21] Lee K. P. (2015). Psycholosocial factors associated with psychological insulin resistance in primary care patients in Hong Kong. *Journal of Clinical & Translational Endocrinology*, 2(4), 157–162. 10.1016/j.jcte.2015.10.001 29159120 PMC5685026

[bib22] Monnier L. Lapinski H. & Colette C. (2003). Contributions of fasting and postprandial plasma glucose increments to the overall diurnal hyperglycemia of type 2 diabetic patients: Variations with increasing levels of HbA_1c_. *Diabetes Care*, 26(3), 881–885. 10.2337/diacare.26.3.881 12610053

[bib23] Netemeyer R. G. Bearden W. O. & Sharma S. (2003). *Scaling procedures: Issues and applications* (1st ed.). Thousand Oaks, CA: Sage.

[bib24] Nichols G. A. Rosales A. G. Perrin N. A. & Fortmann S. P. (2014). The association between different A1C-based measures of glycemia and risk of cardiovascular disease hospitalization. *Diabetes Care*, 37(1), 167–172. 10.2337/dc13-1300 23990520 PMC3867992

[bib25] Petrak F. Stridde E. Leverkus F. Crispin A. A. Forst T. & Pfützner A. (2007). Development and validation of a new measure to evaluate psychological resistance to insulin treatment. *Diabetes Care*, 30(9), 2199–2204. 10.2337/dc06-2042 17575092

[bib26] Peyrot M. Barnett A. H. Meneghini L. F. & Schumm-Draeger P. M. (2012). Insulin adherence behaviours and barriers in the multinational Global Attitudes of Patients and Physicians in Insulin Therapy study. *Diabetic Medicine*, 29(5), 682–689. 10.1111/j.1464-5491.2012.03605.x 22313123 PMC3433794

[bib27] Polinski J. M. Smith B. F. Curtis B. H. Seeger J. D. Choudhry N. K. Connolly J. G. & Shrank W. H. (2013). Barriers to insulin progression among patients with type 2 diabetes: A systematic review. *The Diabetes Educator*, 39(1), 53–65. 10.1177/0145721712467696 23192599

[bib28] Polonsky W. H. Hajos T. R. S. Dain M. P. & Snoek F. J. (2011). Are patients with type 2 diabetes reluctant to start insulin therapy? An examination of the scope and underpinnings of psychological insulin resistance in a large, international population. *Current Medical Research and Opinion*, 27(6), 1169–1174. 10.1185/03007995.2011.573623 21469914

[bib29] Prochaska J. O. (1994). Strong and weak principles for progressing from precontemplation to action on the basis of twelve problem behaviors. *Health Psychology*, 13(1), 47–51. 10.1037/0278-6133.13.1.47 8168471

[bib30] Prochaska J. O. Redding C. A. & Evers K. E. (2008). The transtheoretical model and stages of change. In Glanz K. Rimer B. K. & Viswanath K. (Eds.), *Health behavior and health education: Theory, research, and practice* (4th ed., pp. 97–121). San Francisco, CA:Jossey-Bass.

[bib31] Rosner B. (2006). *Fundamentals of biostatistics* (6th ed.). Belmont, CA: Thomson Brooks/Cole.

[bib32] Shtaynberger J. & Krebs P. (2016). Associations between decisional balance and health behaviors among adult cancer survivors. *Journal of Cancer Education*, 31(4), 749–754. 10.1007/s13187-016-1097-z 27524376 PMC5073008

[bib33] Snoek F. J. Skovlund S. E. & Pouwer F. (2007). Development and validation of the insulin treatment appraisal scale (ITAS) in patients with type 2 diabetes. *Health and Quality of Life Outcomes*, 5, 69 10.1186/1477-7525-5-69 18096074 PMC2241589

[bib34] Therawiwat M. Imamee N. & Khamklueng T. (2013). Self-efficacy, decisional balance and stages of change on dietary practices among metabolic syndrome persons, Uthai Thani Province. *Journal of the Medical Association of Thailand*, 96(5, Suppl), S131–S137. 24851583

[bib35] Tong W. T. Vethakkan S. R. & Ng C. J. (2015). Why do some people with type 2 diabetes who are using insulin have poor glycaemic control? A qualitative study. *BMJ Open*, 5(1), e006407 10.1136/bmjopen-2014-006407 PMC4316456 25633285

[bib36] Ullman J. B. (2006). Structural equation modeling: Reviewing the basics and moving forward. *Journal of Personality Assessment*, 87(1), 35–50. 10.1207/s15327752jpa8701_03 16856785

[bib37] Waltz C. F. Strickland O. & Lenz E. R. (1991). *Measurement in nursing research* (2nd ed.). Philadelphia, PA: F. A. Davis.

[bib38] Wang H. F. & Yeh M. C. (2012). Psychological resistance to insulin therapy in adults with type 2 diabetes: Mixed-method systematic review. *Journal of Advanced Nursing*, 68(4), 743–757. 10.1111/j.1365-2648.2011.05853.x 22050365

[bib39] Woudenberg Y. J. C. Lucas C. Latour C. & Scholte op Reimer W. J. M. (2012). Acceptance of insulin therapy: A long shot? Psychological insulin resistance in primary care. *Diabetic Medicine*, 29(6), 796–802. 10.1111/j.1464-5491.2011.03552.x 22150962

[bib40] Yavuz D. G. Ozcan S. & Deyneli O. (2015). Adherence to insulin treatment in insulin-naive type 2 diabetic patients initiated on different insulin regimens. *Patient Preference and Adherence*, 9, 1225–1231. 10.2147/PPA.S87935 26346988 PMC4556254

[bib41] Yu N. C. Su H. Y. Chiou S. T. Yeh M. C. Yeh S. W. Tzeng M. S. & Sheu W. H. H. (2013). Trends of ABC control 2006-2011: A National Survey of Diabetes Health Promotion Institutes in Taiwan. *Diabetes Research and Clinical Practice*, 99(2), 112–119. 10.1016/j.diabres.2012.11.018 23265923

